# Improvement, Validation, and Analysis of Colles Fracture Treated with an Integrated Retainer Pad Splint

**DOI:** 10.1155/2022/8461995

**Published:** 2022-11-10

**Authors:** Xianyu Meng, Fengjiu Li, Xu Zhang, Donghui Zhao, Quan Li, Qiuhong Li, Lei Wang, Ji Yang, Lijie Dong, Wei Jiang

**Affiliations:** ^1^The First Affiliated Hospital, Heilongjiang University of Chinese Medicine, Harbin, China; ^2^Heilongjiang University of Chinese Medicine, Harbin 150040, China; ^3^Heilongjiang Center for Disease Control and Prevention, Harbin, China

## Abstract

**Objective:**

To observe the effectiveness and safety of the Colles fracture treated with the integrated retainer pad splint and to compare the clinical and radiological outcomes of the integrated retainer pad splint and the traditional bamboo curtain splint in the treatment of the Colles fracture.

**Methods:**

A total of 100 patients with Colles fractures were randomly divided into two groups: the treatment group was fixed with the integrated retainer pad splint (IS), and the control group was fixed with the traditional bamboo curtain splint (TS).The range of wrist motion was measured at follow-up examinations, and volar inclination, ulnar deviation, and radial height were measured on radiographs. Regular follow-up wrist imaging examinations and functional examinations were performed before reduction, after reduction, and at the 1st, 3rd, 5^th^, and 8th weeks. The two groups were compared in terms of convenience, fracture healing time, X-ray data of volar inclination, ulnar deviation, radial height, and wrist joint function. The relevant data were analyzed with SPSS 25.0 statistical software.

**Results:**

There were no notable differences in gender, age, and injured side between IS and TS groups. In terms of operation time, IS was better than the TS group (*P* < 0.05), and the operation time in the IS group was shorter. On the basis of X-ray data of volar inclination, ulnar deviation, and radial height measured on radiographs, the difference between the IS and TS groups was statistically significant (*P* < 0.05), which showed that the IS group was more stable in fracture fixation and had less reduction loss during the treatment process. At the 8th week of treatment, the wrist Gartland–Werley score of the two groups showed that the two fixation methods are equivalent in restoring wrist joint function (*P* > 0.05); however, in terms of the excellent and good rate of wrist joint function, the IS group scored 96% was higher than the TS group (80%).

**Conclusion:**

Compared with the traditional bamboo curtain splint, the integrated retainer pad splint is more convenient and stable, and it has less reduction loss during the treatment. Repair of the Colles fracture using the integrated retainer pad splint with external fixation results in nearly normal return of function, which is significantly better than using the traditional bamboo curtain splint.

## 1. Introduction

Fractures of the distal radius are one of the most common types of fractures seen in clinics, accounting for 17% of all body fractures [1]. Especially the extension fracture of the distal radius, known as the Colles fracture, has a very high incidence in northeast China due to the accelerated aging of China's population and the cold slippery roads in winter in northeast China. In most cases, these fractures occur more commonly in women than in men, increase in frequency with progressing age, and are often the result of low-energy falls [2].

Generally, most Colles fractures could be treated nonoperatively and achieve satisfactory therapeutic effects, and the fracture could be reset with manual closed reduction and kept immobilized in good alignment with external fixation, but they have received little attention in the literature [3]. In the external fixation scheme, the sugar tong gypsum and short arm cast are the most commonly used option after reduction, but the resulting inadequate immobilization is a risk factor for the loss of reduction, requiring either repeat reduction or surgical treatment [4]. It is generally believed that gypsum fixation is often associated with residual deformity, stiffness, pain, and swelling [5]. In patients who have significant hand and wrist swelling, the traditional bamboo curtain splint should be used rather than gypsum [6]. The traditional bamboo curtain splint has the advantages of being randomly adjustable, having good air permeability, and having high X-ray transmission compared with the gypsum, and it is characterized by “simplicity, convenience, and effectivity” and can not only avoid surgical trauma complications but also can be easily accepted by patients. However, the traditional bamboo curtain splint has shortcomings such as too many components and complicated steps. Therefore, it is evidently important to develop a kind of splint that not only retains the advantages but also avoids the disadvantages of the traditional bamboo curtain splint. There has been no study previously published regarding the improvement of splints that specifically focused on the integration of various components. In clinical practice, with the approval of the Heilongjiang University of Chinese Medicine Ethics Committee, we applied the integrated retainer pad splint and achieved excellent curative effects to treat the Colles fracture, which is described as follows.

## 2. Patients and Methods

### 2.1. Subjects

This study included 100 patients with Colles fractures diagnosed and treated in our hospital from December 2010 to December 2021. A randomized controlled study was conducted that was divided into a treatment group and a control group. Reduction and external fixation were performed by the same chief physician. The treatment group was fixed with the integrated retainer pad splint (IS) of the distal radius, while the control group was fixed with the traditional bamboo curtain splint (TS).

### 2.2. Diagnostic Criteria

The Colles fracture diagnostic criteria [7] in Green's Operative Hand Surgery were adopted: (1) to clarify the history of trauma; (2) the wrist swelling, pain, palpable bone rubbing, and abnormal activities; (3) the activity dysfunction or loss; (4) the anteroposterior bayonet deformity and lateral fork deformity; (5) the radiographic X-ray or CT features of the distal radius fracture composed of dorsal comminution, dorsal angulation, dorsal displacement, radial shortening, and an associated fracture of the ulnar styloid.

### 2.3. Inclusion Criteria

(1) Meet the above diagnostic criteria of volar inclination <0° and ulnar deviation <10°. (2) The reduction reached the standard of volar inclination 10 ∼ 15°, ulnar deviation 20 ∼ 25°, and wrist deformity disappearance [8].

### 2.4. Exclusion Criteria

(1) Combined with fractures in other parts or injuries of blood vessels, nerves, and tendons; (2) people with mental illness and uncontrollable behavior; (3) an open fracture; patients who met any of the exclusion criteria were excluded from the study.

### 2.5. Methods

#### 2.5.1. Treatment Methods

**Figure 1:**
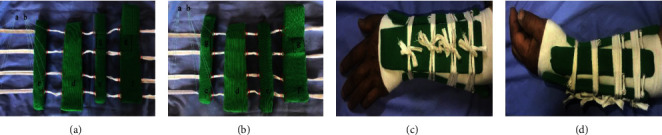
The composition and application of an integrated retainer pad splint for the distal radius in the treatment group. (a) Integrated retainer pad splint picture of real products, left wrist; (b) Integrated retainer pad splint picture of real products, right wrist; (c) dorsal view of the Colles fracture of the left wrist with an integrated retainer pad splint; (d) lateral view of the Colles fracture of the left wrist with an integrated retainer pad splint; (A) a cotton tape through the four splints; (B) splint drilling and threading; (C) arc of the ulnar splint is 20°; (D) arc of the palm splint is 15°; (E) arc of the radial splint is 20°; (F) arc of the dorsal splint is 15°; (G) pastable pressure pad.

Treatment group: fracture reduction completed, the treatment group was placed with the integrated retainer pad splint at the distal radius and tightened the four cotton tapes in turn in a cross direction, tied the knot on the dorsolateral side, and suspended the injured limb at the neutral position of the forearm at 90° elbow flexion. The forearm was in a neutral position, with the palm attached to the chest, as shown in Figure 1.

**Figure 2:**
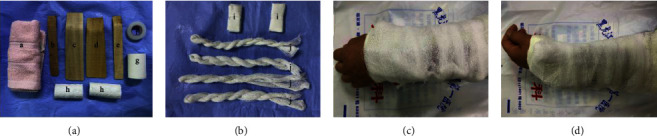
Composition and application of the traditional bamboo curtain splint in the control group. (a) Traditional bamboo curtain splint requires spare dispersing components; (b) traditional bamboo curtain splint requires improvised components; (c) dorsal view of the Colles fracture of the left wrist with a traditional bamboo curtain splint; (d) lateral view of the Colles fracture of the left wrist with a traditional bamboo curtain splint; (A) cotton liner; (B) ulnar side splint; (C) dorsal side splint; (D) volar side splint; (E) radial side splint; (F) adhesive tape for the skin; (G) adhesive plaster; (H) bandages; (I) two pressure pads; (J) four cotton tapes.

Control group: fracture reduction completed, and in the control group, two pads were made with adhesive tape and cotton cloth, and two pressure pads were glued and fixed on the dorsal and radial sides of the distal fracture end with an adhesive tape. The middle and lower forearm and wrist were wrapped with the cotton liner. We placed the traditional bamboo curtain splint in sequence on the dorsal, volar, radial, and ulnar sides, and where the dorsal and radial bamboo curtain splint exceeded the wrist joint, the volar and ulnar bamboo curtain splint were lower than the wrist joint. The assistant held four bamboo curtain splints with both hands; another assistant tied the bamboo curtain splints with four cotton tapes. The cotton tapes were wound around the surface of the bamboo curtain splint and then tied in a dorsolateral knot. The tightness is that the cotton tape can move up and down 1 cm on the surface of the bamboo curtain splint. Further we wrapped and secured with bandages, and we suspended the injured limb at the neutral position of the forearm at 90° elbow flexion; the forearm was in a neutral position, with the palm attached to the chest, as shown in Figure 2.

#### 2.5.2. Efficacy Assessment Methods

Data collection: the blood circulation and sensory and motor status of the affected limb were examined after external fixation. Follow-up visits were made immediately after reduction and fixation, at the 1st, 3rd, 5^th^, and 8th weeks, and external fixation was removed at the 5th week. We record the time required for two groups of patients with external fixation to evaluate the convenience of using a splint, measuring volar inclination, ulnar deviation, radial height, and the wrist joint function score at the 8th week.

### 2.6. Statistical Methods

SPSS25.0 statistical software was used for data statistical analysis.

## 3. Results

Through the comparative study of the treatment of the Colles fracture with the integrated retainer pad splint and the traditional bamboo curtain splint, the operation time of the integrated retainer pad splint was less than two minutes, which was more efficient and shorter than the traditional bamboo curtain splint. In terms of the X-ray data of the 1st, 3^rd^, and 5th weeks, the fixation of the integrated retainer pad splint was more stable, and the reduction loss was less during the treatment. In Gartland–Werley wrist function at the 8th week of treatment, the rate of good results with the integrated retainer pad splint was higher than the traditional bamboo curtain splint for the distal radius.

### 3.1. Comparison of Gender, Age, and the Injured Side of Patients between the Two Groups (Table 1)

As shown in Table 1, the comparison of gender, age, and the injured side between the treatment group and the control group was *P* > 0.05, indicating comparability between the two groups. 

### 3.2. Comparison of Splint Operation Time and Healing Time between the Two Groups (Table 2)

As shown in Table 2, the comparison of the operation time of splint fixation between the two groups showed a statistically significant difference (*P* < 0.05). In terms of the convenience of splint use, the treatment group was significantly better than the control group. There was no significant difference in fracture healing time between the two groups (*P* > 0.05), and there was no significant difference between the two fixation methods on fracture healing time. 

### 3.3. Comparison of Imaging Data Results between the Two Groups (Table 3)

As shown in Table 3, comparison of volar inclination, ulnar deviation, and radial height between the two groups before and after treatment (*P* > 0.05) indicates comparability between the two groups. At the 1st, 3rd, 5^th^, and 8th weeks of treatment, volar inclination, ulnar deviation, and radial height were compared between the groups (*P* < 0.05), and the treatment group had more stable fracture fixation and less reduction loss during treatment.

### 3.4. Gartland–Werley Wrist Function Score [9] Comparison between the Two Groups (Table 4)

As shown in Table 4, there was no statistical significance in Gartland–Werley wrist function scores between the two groups at the 8th week (*P* > 0.05). The excellent and good rate of wrist function in the treatment group was higher than that in the control group. 

### 3.5. X-Ray Films of Typical Cases with the Integrated Retainer Pad Splints Were Used

**Figure 3:**
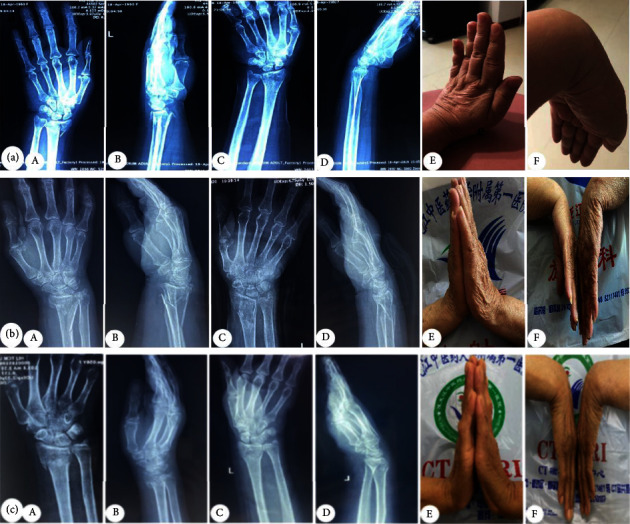
Anteroposterior and lateral radiographs of the Colles fracture before and after reduction show that volar inclination and ulnar deviation returned to normal, and wrist deformity disappeared. Using the integrated retainer pad splint of the distal radius, the radial height, volar inclination, and ulnar deviation of the wrist were maintained easily and effectively. (a) Typical case 1; (b) typical case 2; (c) typical case 3; (A) anteroposterior radiograph before reduction; (B) lateral radiograph before reduction; (C) anteroposterior radiograph after reduction; (D) lateral radiograph after reduction; (E) wrist dorsiflexion function at the 8th week follow-up; (F) wrist palmar flexion function at the 8th week follow-up.

This is shown in Figure 3.

## 4. Discussion

As an important part of Chinese medicine, the small splint plays an important role in the treatment of traditional Chinese orthopedics. In the Qin and Han Dynasties, (Chinese Tibetan Sutra) recorded that “a large section of the injured limb was sandwiched with bamboo slices.” Ge Hong in the Jin Dynasty (Handbook of Prescriptions for Emergencies) described the manipulation and fixation of small splints. Ming Dynasty, Zhu Su (Prescriptions for Universal Relief), “Bend the palm outward to keep the order straight,” the extension fracture of the distal radius was described for the first time. The traditional bamboo curtain splint is the application of continuous clinical experience summarized by the orthopedics department of traditional Chinese medicine, reflecting the principles of “combination of dynamic and static, equal emphasis on muscles and bones, internal and external treatment, and doctor-patient cooperation” [10]. Under the treatment principle, we made the integrated retainer pad splint by integrating various parts of the traditional bamboo curtain splint.

In the process of conservative treatment of the Colles fracture, external fixation is an important link, and whether the external fixation method is simple and effective is crucial [11, 12]. The traditional bamboo curtain splint, composed of four splints, two pressure pads, and four cotton tapes, is an important treatment method for the Colles fracture because it is more portable, continuously adjustable, easy to disassemble, highly permeable, and X-ray transmittable than gypsum [13]. The pressure pads and bamboo splints not only do not irritate the skin but also have ductility and elasticity to maintain a certain support form. However, in clinical use, it was found that the parts of the traditional bamboo curtain splint were scattered and not matching, the two pressure pads, four splints and four cotton tapes needed to be made temporarily during use, and the pressure pads were made temporarily and then pasted. If the external splint operating time took too long, the probability of fracture displacement increased [14]. As swelling lessens, splint fixation loosens, and the cotton tapes slide distally, fracture fixation becomes unstable and must be adjusted by frequent return visits. Therefore, it is necessary to improve.

By improving the traditional bamboo curtain splint, the integrated retainer pad splint integrates four bamboo curtain splints, two pressure pads, and four binding cotton tapes, which can provide a relatively stable fixation state and effectively maintain a good correspondence after the reduction of the fracture end. Clinicians no longer need to temporarily make and paste the pressure pad, nor do they need to make the binding cotton tape; they just need to directly bind the splint, which improves convenience and significantly shortens the splint fixation time. At the 1st, 3^rd^, and 5th weeks of follow-up, the integrated retainer pad splint compared with the traditional bamboo curtain splint was more stable and caused less loss of reduction in the treatment process. The cotton tapes of the integrated retainer pad splint passed through the horizontal hole in the middle of the four splints, will not slide to the distal end because of swelling regression and muscle contraction during functional exercise, and the cotton tape is not easy to loosen in the process of fixation, so the fixation of fractures is more stable. The integrated retainer pad splint not only reserved the light material, good air permeability, low price, convenient disassembly, and good X-ray transmission advantages of the traditional bamboo curtain splint but also significantly improved the use convenience and fixed stability, and the rate of excellent and good treatment naturally increased.

In the course of treatment, attention should be paid to factors such as volar inclination, ulnar deviation, and radial height, which may lead to fracture displacement and external fixation failure and are also evaluation standards for fracture reduction [15, 16]. We present results of the integrated retainer pad splint which was developed in China for the treatment of distal fractures of the radius. After reduction, the integrated retainer pad splint was applied, the pressure pad was placed at the fractured end according to the fracture displacement direction, the splint gaps were adjusted according to the circumference of the limb, and the cotton tapes were adjusted until the cotton tapes could be moved up and down by 1 cm. The mechanism of the integrated retainer pad splint was external force, internal reaction force, and a limb placement pattern opposite to the displacement direction. After fracture reduction, the tendency to displace is still present, and the integrated retainer pad splint keeps the limb against the direction of the trauma mechanism to avoid the possibility of fracture redisplacement. The binding force of the integrated retainer pad splint is the source of local external fixation force. During continuous fixation, this continuous binding can be applied to the fracture by pressing the pad, further correcting the residual displacement of the fracture. The longitudinal contraction movement of the muscles makes the two fracture ends produce longitudinal extrusion pressure, strengthening the close contact of the fracture ends and increasing stability. The external fixation pressure of the integrated retainer pad splint on the fracture end of the limb and the expansion of the circumference of the limb during muscle contraction produce an internal reaction force that reaches a dynamic balance. The patient should, whenever possible, move the distal joints of the limbs and actively engage in muscle contraction and diastolic exercises.

The traditional bamboo curtain splint (TS), pressure pads, and bindings are made onsite, and the material cost is about 50 yuan RMB ($7∼8). The integrated retainer pad splint (IS) is developed and mass-produced by ourselves, and each one costs 10 yuan RMB ($1∼2). The integral retainer pad splint can eliminate the factors that may cause redisplacement after reduction. Patients should be encouraged to keep their hands elevated and actively flex and extend their fingers. “Combination of dynamic and static,” “Equal emphasis on muscles and bones,” the integral retainer pad splint allows maximum movement of the affected limb while maintaining the stable reduction of the fracture end. “Internal and external treatment,” “Doctor-patient cooperation” during the application of the integral retainer pad splint to treat the Colles fracture led to different treatment methods being adopted according to different fracture periods. It is emphasized that in the process of using the integral retainer pad splint, the patient should cooperate with the doctor for treatment and adjust the integrated retainer splint timely according to the doctor's advice.

## 5. Conclusion

To sum up, the integrated retainer pad splint embodies stable fixation and early motion and is a representative method for treating the Colles fracture in the orthopedics department of traditional Chinese medicine that is safe, advanced, portable, practical, and suitable for clinical promotion.

## Figures and Tables

**Table 1:** Comparison of gender, age, and the injured side ($\bar{x}\pm s$).

Group	Case number	Gender		Age ($\bar{x}\pm s$)	Injured side (case)	
		Man	Woman		Left	Right
Treatment group	50	7	43	64.30 ± 6.25	22	28
Control group	50	8	42	67.28 ± 6.67	20	30
Statistics of the test		*χ * ^2^ = 0.525		*t* = −0.530	*χ * ^2^ = 0.556	
*P* value		0.409		0.512	0.456	

**Table 2:** Comparison of splint operation time and healing time ($\bar{x}\pm s$).

Group	Operation time (m)	Fracture healing time (d)
Treatment group	2.66 ± 1.33	33.23 ± 2.26
Control group	8.58 ± 2.51	34.33 ± 2.45
Statistics of the test	*t* = −23.278	*t* = −0.436
*P* value	0.000	0.646

**Table 3:** Comparison of image data results ($\bar{x}\pm s$).

Time	Radial inclination angle (°)		Ulnar angle (°)		Radial height (mm)	
	Treatment group	Control group	Treatment group	Control group	Treatment group	Control group
Before reduction	−14.25 ± 3.22	−11.26 ± 3.98	12.75 ± 3.25	13.32 ± 1.89	2.82 ± 3.88	2.02 ± 2.32
*P* value	0.165		0.152		0.520	
After reduction	11.40 ± 0.96	11.33 ± 0.85	21.95 ± 0.65	19.76 ± 0.89	11.73 ± 1.08	11.88 ± 0.88
*P* value	0.811		0.480		0.667	
3 weeks	10.75 ± 0.57	11.19 ± 0.80	20.32 ± 0.56	19.20 ± 0.20	11.40 ± 0.89	9.57 ± 0.57
*P* value	0.032		0.023		0.018	
5 weeks	10.43 ± 0.82	9.68 ± 0.92	20.15 ± 0.53	19.32 ± 0.23	11.28 ± 0.94	9.56 ± 1.73
*P* value	0.030		0.024		0.016	
8 weeks	10.87 ± 0.85	9.78 ± 0.85	20.35 ± 0.73	19.10 ± 0.20	11.20 ± 0.95	9.57 ± 1.72
*P* value	0.026		0.021		0.018	

**Table 4:** Comparison of the Gartland–Werley wrist function at the 8th week follow-up.

Group	Case number	Wrist function score (example)				Excellent and good rate (%)
		Excellent	Good	Fair	Poor	
Treatment group	50	40	8	2	0	96
Control group	50	33	7	8	2	80
*P* value	0.321					

## Data Availability

The data that support the findings of this study are available from the corresponding author upon reasonable request.
